# A Putative Cell Surface Receptor for White Spot Syndrome Virus Is a Member of a Transporter Superfamily

**DOI:** 10.1371/journal.pone.0033216

**Published:** 2012-03-13

**Authors:** Huai-Ting Huang, Jiann-Horng Leu, Po-Yu Huang, Li-Li Chen

**Affiliations:** 1 Institute of Marine Biology, National Taiwan Ocean University, Jhongjheng District, Keelung City, Taiwan, Republic of China; 2 Center of Excellence for Marine Bioenvironment and Biotechnology, National Taiwan Ocean University, Jhongjheng District, Keelung City, Taiwan, Republic of China; German Primate Center, Germany

## Abstract

White spot syndrome virus (WSSV), a large enveloped DNA virus, can cause the most serious viral disease in shrimp and has a wide host range among crustaceans. In this study, we identified a surface protein, named glucose transporter 1 (Glut1), which could also interact with WSSV envelope protein, VP53A. Sequence analysis revealed that Glut1 is a member of a large superfamily of transporters and that it is most closely related to evolutionary branches of this superfamily, branches that function to transport this sugar. Tissue tropism analysis showed that *Glut1* was constitutive and highly expressed in almost all organs. Glut1's localization in shrimp cells was further verified and so was its interaction with *Penaeus monodon* chitin-binding protein (PmCBP), which was itself identified to interact with an envelope protein complex formed by 11 WSSV envelope proteins. *In vitro* and *in vivo* neutralization experiments using synthetic peptide contained WSSV binding domain (WBD) showed that the WBD peptide could inhibit WSSV infection in primary cultured hemocytes and delay the mortality in shrimps challenged with WSSV. These findings have important implications for our understanding of WSSV entry.

## Introduction

White spot syndrome virus (WSSV) is the causative agent of a disease that has led to severe mortality rates of cultured shrimps in Taiwan and many other countries. WSSV, a kind of large enveloped DNA virus, has a wide host range among crustaceans [1], [2]. After the sequences of the WSSV genome for various isolates have been revealed, research regarding protein-protein interaction between shrimp and virus, shrimp itself or virus itself are now taken into consideration [3], [4], [5]. Above all, the interaction between the receptor/co-receptor of the host cell and the receptor-binding protein of virus is highly remarkable because binding and entry of viruses requires specific interactions between the structural proteins on the virus and cell surface receptor complexes on target cells. The molecules to which viruses bind constitute a diverse collection of cellular proteins, carbohydrates, and lipids. They differ from one virus to the next, and they range from abundant and ubiquitous to rare and cell specific [6]. To date, more than one shrimp protein was supposed to participate in WSSV infection [7], [8], [9], [10]. However, there is no further evidence to verify whether these host proteins cooperate with each other to mediate virus infection or the exact functions these proteins play while infecting. We still can not precisely illustrate the process how WSSV enters the host cell.

Key issues in virology have been identification of cell-surface virus receptor, determination of receptor expression patterns, and elucidation of the effects of infection on the normal functions of the molecules [11]. In the previous study, a host membrane protein, *Penaeus monodon* chitin-binding protein (PmCBP), which can specifically interact with WSSV envelope protein VP53A, was identified [8]. The data showed that *in vivo* neutralization using recombinant VP53A and PmCBP can reduce and delay mortality upon WSSV challenge, indicating that PmCBP was involved in WSSV infection. Moreover, besides VP53A, PmCBP was found to at least interact with ten other envelope proteins (VP24, VP110, VP53B, VP337, VP32, VP124, VP41A, VP51B, VP60A and VP39B) [10]. These findings suggest that the process of WSSV infection was extremely complex and that there must still be an unknown number of proteins which play a part.

To continue to unravel the process of WSSV entry and the formation of entry-related complexes, we identified a *P. monodon* and *Litopenaeus vannamei* surface protein, named glucose transporter 1 (Glut1), which could also interact with VP53A. Glut1's localization in shrimp cells was further characterized and its interaction with PmCBP was also verified.

## Results

### Identification of glucose transporter 1 (Glut1) in shrimps

**Figure 1:**
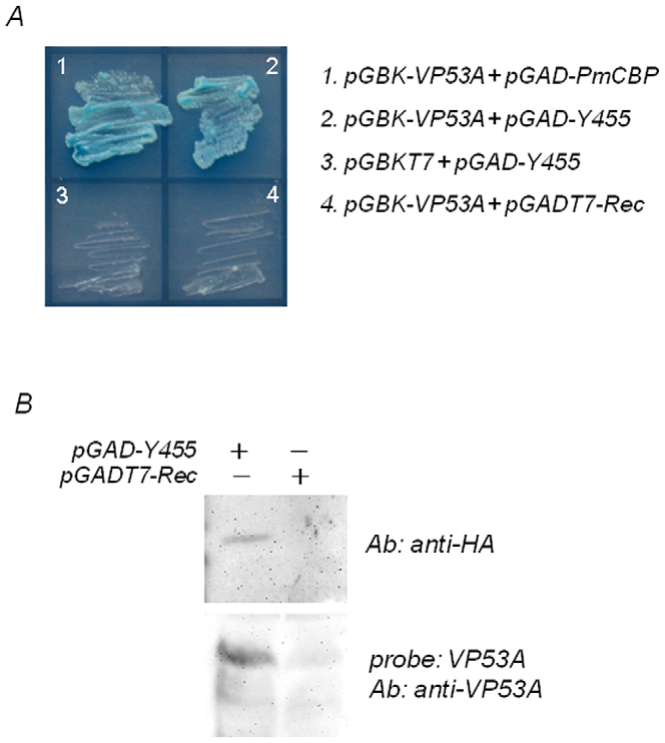
VP53A interacted with Y455 clone in yeast. (A) Yeast representing protein-protein interactions grew on high-stringency (–Leu/–Trp/–His/–Ade) medium. Plasmids used in this figure are described in the Materials and Methods. The blue signal in image was due to the presence of X-α-Gal. (B) Far western blot. Upper image: the yeast lysates were transferred on PVDF membrane and then detected with anti-HA antibody. Lower image: the yeast lysates were transferred on PVDF membrane, incubated with recombinant VP53A and the detected with anti-VP53A antibody. The yeast transformed with pGADT7-Rec only was set as control.

To identify shrimp proteins that bind WSSV envelope protein VP53A, we performed a yeast two-hybrid screen with VP53A as bait and the library was constructed from shrimp *Penaeus monodon* [8]. In the previous study, the screening of the cDNA library led to isolate a membrane protein named *P. monodon* chitin-binding protein (PmCBP). In this study, as shown in Fig. 1A, a positive clone Y455 which coded 106 amino acids was identified. Yeast expressing BD-contained VP53A and AD-contained Y455 formed colonies on SD medium lacking leucine (Leu), tryptophan (Trp), histidine (His), and adenine (Ade). The results suggested strongly that, in yeast, VP53A and Y455 interact, and that examination of this interaction under different conditions was warranted. The interaction between VP53A and the gene product form clone Y455 was further confirmed by far western blotting. As shown in Fig. 1B, the gene product from clone Y455 containing HA tag can be specific detected with anti-HA antibody. After incubating with recombinant VP53A, the gene product can be detected with anti-VP53A antibody. The result indicated that VP53A interacts with the gene product of clone Y455.

**Figure 2:**
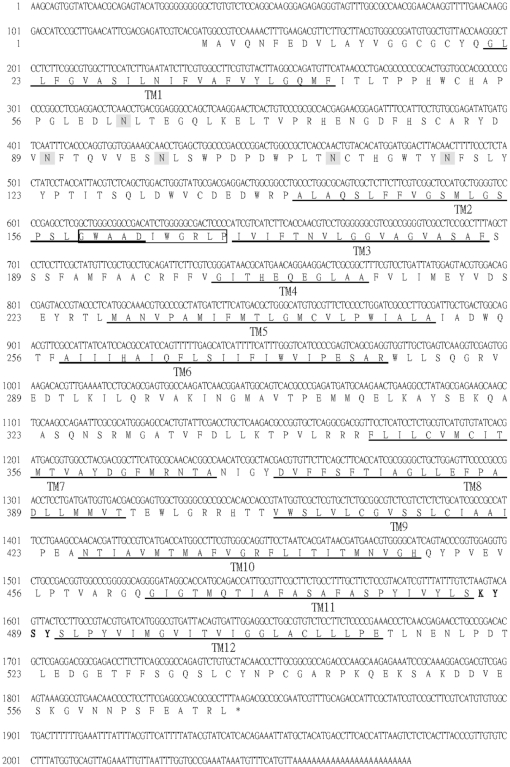
Primary structure of Y455 (Glut1) and major structural features. The gene product of full-length of Glut1 contains 569 amino acids with 12 hydrophobic potential TM sequences. TM sequences are identified by the Kyte-Doolittle algorithm (Kyte and Doolittle, 1982) and are indicated by underlining the amino acid sequence. Signature sequences for MFS transporter are boxed. Potential N-linked glycosylation sites are shown in shaded boxes. The putative WSSV binding domain (WBD) between TM11 and TM12 is shown in bold font.

Because the clone Y455 only contained the last 106 amino acids of the 3′ terminus of an unknown gene, the 5′ terminus of this gene was obtained by the method of rapid amplification of the cDNA 5′ end (5′ RACE). In this study, *Litopenaeus vannamei Glut1*, not *P. monodon Glut1*, was fully sequenced because *L. vannamei* was the experimental species in the further study of gene analysis. Fig. 2 shows the predicted amino acid sequence and major structural features of the full-length of clone Y455. The open reading frame (ORF) encodes a protein of 569 amino acids with a theoretical size of about 63 kDa (and a predicted isoelectric point of 4.96). The nucleotide sequence surrounding the methionine start codon (ACG**ATG**G) of the predicted protein conformed to the Kozak rule of an efficient context for eukaryotic translation initiation [12], [13]. A polyadenylation signal (AATAAA) was located 193 bp downstream of the translational stop codon. The deduced amino acid sequence of the ORF has five NX(S/T) sites for potential N-linked glycosylation. The Kyte-Doolittle program [14] of this ORF suggested the presence of 12 hydrophobic potential transmembrane (TM) sequences, consistent with its possible presence in cellular membranes. The protein contains several dileucines, which may be required for endocytosis [15]. Because the last 106 amino acid interacting with WSSV VP53A contained the short extracellular segment KYSY between TM11 and TM12, a putative WSSV binding domain (WBD) was suggested to locate at this region.

Sequence analysis using NCBI BLAST program (http://www.ncbi.nlm.nih.gov/) revealed that the gene product of full-length of Y455 showed strong homology to a MFS transporter of *Aedes aegypti* (GenBank accession no.: XP_001662291), a cation transporter protein of *Drosophila grimshawi* (GenBank accession no.: XP_001996245), an organic cation transporter protein of *Harpegnathos saltator* (GenBank accession no.: EFN86109), an organic cation transporter of *Apis mellifera* (GenBank accession no.: XP_395856), an organic cation transporter of *Pediculus humanus corporis* (GenBank accession no.: XP_002425801) and an organic cation transporter 1 of *Camponotus floridanus* (GenBank accession no.: EFN71065). Those proteins were categorized to a member of a large superfamily of transporters, major facilitator superfamily (MFS), which generally contain 12 hydrophobic TM sequences [16]. The MFS transporters have been classified into 187 evolutionary branches or subgroups that transport distinct categories of solutes. The MFS signature sequence, which occurs in the hydrophilic loop that separates TM2 and TM3, is the most highly conserved sequence in this transporter superfamily [16], [17]. Analyzing the deduced amino acid sequence of the ORF, the MFS signature sequence was found TM2 and TM3 as in Fig. 2. It was strongly confirmed that the gene product of full-length of Y455 is a member of MFS transporter and it is most closely related to the sugar porter (SP) family consisting over 133 sequenced members derived from bacteria, archaea and eukarya.

**Figure 3:**
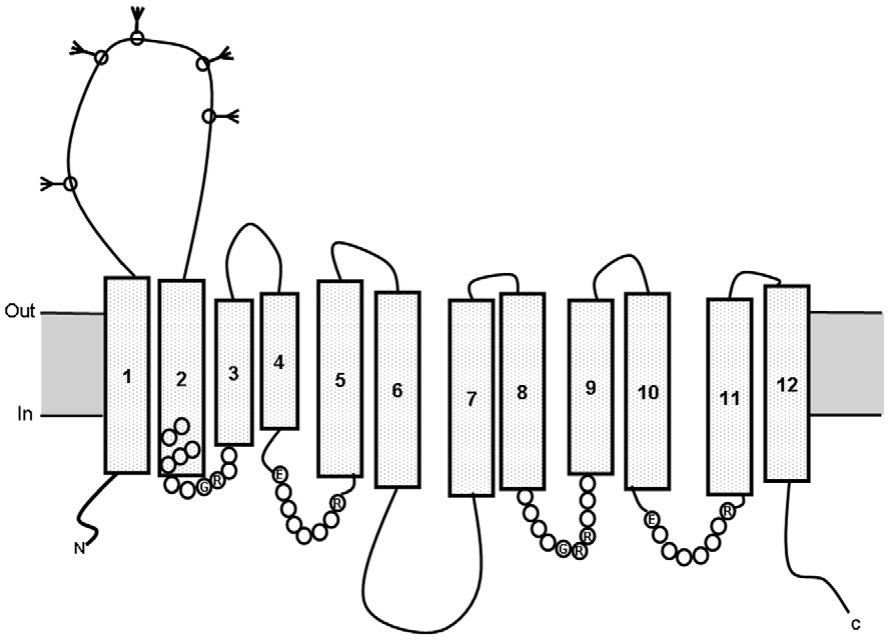
Hypothetcal model for the structure of the gene product of full-length of Y455 (Glut1). The protein was predicted to contain 12 transmembrane (TM) sequences (TM1-12), with both the N- and C-termini intracellularly disposed. N-linked glycosylation can occur in the extracellular loop between TM1 and TM2 as shown. Conserved amino acids are indicated by the appropriate single-letter code; filled circles indicate conservative substitutions. The extracellular portion between TM1 and TM2 was selected to produce the recombinant protein to generate antibody and analyze the protein-protein interaction.

Fig. 3 showed the hypothetical model for the structure of the gene product of full-length of Y455. As reviewed in the paper of glucose transporter [18], the common features revealed by sequence alignment and analysis of the glucose transporters include 12 predicted TM sequences arranged so that both the N- and C-termini are at the cytoplasmic surface. There are large loops between TM1 and TM2 and between helices TM6 and TM7. The large loop between TM6 and TM7 divides the structure into two halves, the N-terminal domain and the C-terminal domain. Conserved motifs in the glucose transporters include GR(R/K) between TM2 and TM3 in the N-terminal half, and correspondingly between TM8 and TM9 in the C-terminal half. Similarly, EXXXXXXR occurs between TM4 and TM5 in the N-terminal half and correspondingly between TM10 and TM11 in the C-terminal half. The N-linked glycosylation can occur in the extracellular loop between TM1 and TM2. It almost completely matched the features of glucose transporter. Thus, the gene product of full-length of Y455 was named as glucose transporter 1 (Glut1).

### Tissue tropism and expression pattern of Glut1

**Figure 4:**
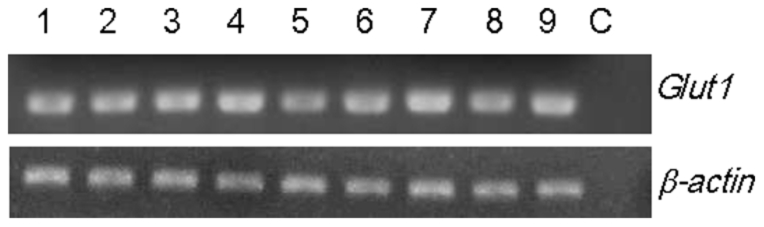
Tissue tropism analysis of *Glut1*. Lane 1–9: PCR amplified *Glut1* and *α-actin* fragments in pleopod, gill, stomach, midgut, heart, lymphoid organ, nervous tissue, hepatopancreas, and hemolymph. C: PCR reaction control which is set up in the same way as the experimental PCRs, but without template DNA added.

RT-PCR was used to reveal the tissue tropism of Glut1. Glut1 was expressed in almost all organs including the pleopod, gill, stomach, midgut, heart, lymphoid organ, nervous tissue, hepatopancreas, and hemolymph (Fig. 4).

### Purification of partial recombinant Glut1 and western blotting

**Figure 5:**
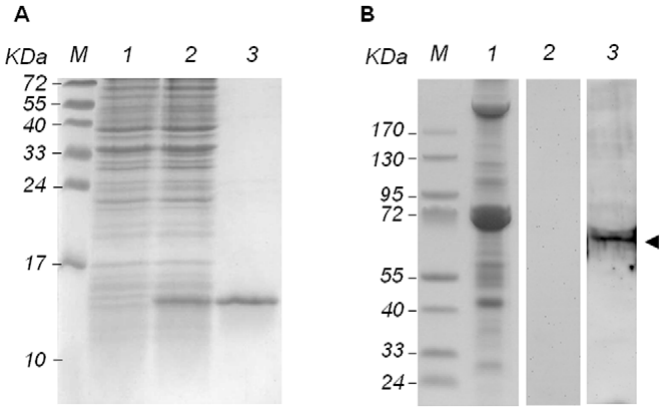
Recombinant expression in *E. coli* purification and western blot of shrimp Glut1. (A) SDS-PAGE of expressed and purified of partial protein. Lanes M, pre-stained molecular weight marker; 1, non-induced; 2, induced; 3, purified Glut1-6×His partial fusion protein. (B) SDS-PAGE and western blotting of Glut1 in heart tissue lysate. Lanes M, pre-stained molecular weight marker; lane 1, coomassie blue stain; lane 2: heart tissue was incubated with rabbit pre-immune antiserum; lane 3: heart tissue was incubated with anti-Glut1 antibody. Glut1 was indicated with arrowhead.

The partial *Glut1* gene, the extracellular region between TM1 and TM2, was expressed in *E. coli* as a 6×His-tagged fusion protein. In the purification process of partial Glut1, after induction with IPTG at 37°C, the SDS-PAGE result showed that a band (about 14 kDa) corresponding to the Glut1-6×His fusion protein was observed in the induced bacterium (Fig. 5A, lane 2). No protein was found at the same position in the non-induced control (Fig. 5A, lane 1). The data indicated that the partial *Glut1* gene was expressed and a purified Glut1-6×His partial fusion protein was obtained (Fig. 5A, lane 3).

Western blotting was performed to detect the Glut1 in *L. vannamei*. Owing to the fact that Glut1 is ubiquitous in a shrimp's body, heart was selected to be tested because it can be easily extracted in buffer. As shown in Fig. 5B, an obvious band about 68 kDa was only detected with Glut1 antibody. No signal was observed in the heart cells treated with rabbit pre-immune antiserum. It indicated that the real molecular weight of Glut1 in shrimp tissue was 68 kDa. It was larger than the theoretical size (63 kDa) owing to the protein modification.

### Cellular localization of Glut1

**Figure 6:**
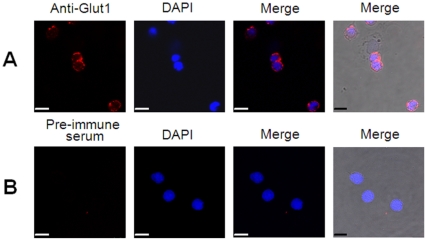
Cellular localizations of Glut1 in *L. vannamei* hemocytes by confocal microscopy. (A) Hemocytes were treated with rabbit anti-Glut1 antibody. (B) Hemocytes were treated with rabbit pre-immune antiserum. Scale bar: 7.5 µm.

Since Glut1 was supposed to be a transporter protein and must be located on the cell surface, hemocytes were applied to identify the speculation *in vivo*. The hemocyte cells withdrawn from *L. vannamei* were fixed, treated with rabbit anti-Glut1 antibody, and analyzed by confocal microscopy. As shown in Fig. 6A, strong fluorescent signals from Glut1 were observed in the plasma membrane. No fluorescent signal was observed in the hemocyte cells treated with rabbit pre-immune antiserum (Fig. 6B). Clearly, this confirmed that Glut1 was a membrane protein.

### Glut1 interacted with PmCBP

**Figure 7:**
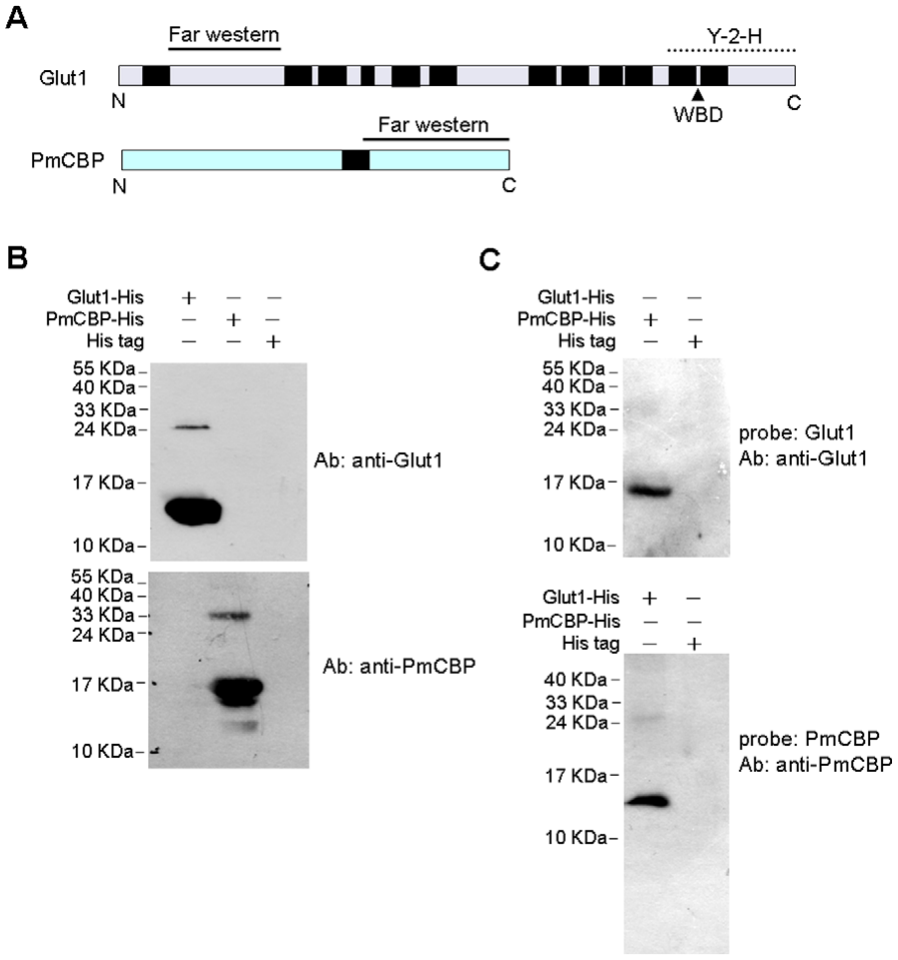
Protein-protein interaction between Glut1 and PmCBP. (A) Schematic diagram of Glut1 and PmCBP. Transmenbrane regions are shown in black boxes. The two solid lines indicated the portions of Glut1 and PmCBP for far western blot. The dot line indicated the portion interacting with VP53A in the yeast-2-hybrid (Y-2-H) assay. (B) SDS-PAGE analysis of the specificity of anti-Glut1 and anti-PmCBP antibodies. The short His tag containing protein purified from *E. coli* transformed with pET28 only was set as control. (C) Far western blot. Upper image: the recombinant PmCBP was transferred on PVDF membrane and then incubated with recombinant Glut1. Lower image: the recombinant Glut1 was transferred on PVDF membrane and then incubated with recombinant PmCBP.

Since the interaction between VP53A and PmCBP has already been revealed [8], the next question, whether Glut1 could interact with PmCBP, was taken into consideration. To confirm this, we carried out an *in vitro* far western blot using the extracellular portion of Glut1 and also the extracellular portion of PmCBP. The schematic diagram of Glut1 and PmCBP is shown in Fig. 7A. As shown in Fig. 7B, anti-Glut1 antibody could capture Glut1 but not PmCBP, and anti-PmCBP antibody could capture PmCBP but not Glut1. It indicated that the specificity of anti-Glut1 and anti-PmCBP antibodies were reliable. In the far western experiments (Fig. 7C), the PmCBP incubated with recombinant Glut1 could be recognized by anti-Glut1 antibody, and the Glut1 incubated with recombinant PmCBP could be recognized by anti-PmCBP antibody. The result strongly suggested that Glut1 could interact with PmCBP at both of their extracellular portions.

### *In vitro* and *in vivo* neutralization

**Figure 8:**
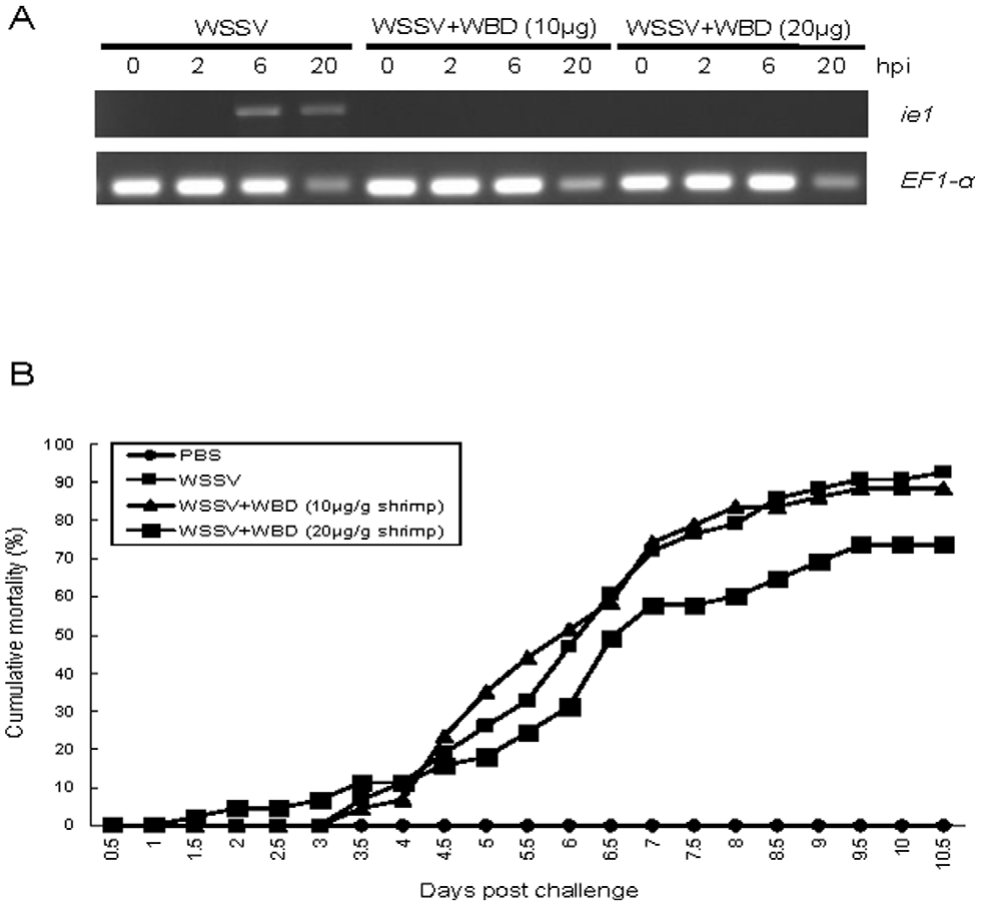
*In vitro* and *in vivo* neutralization. (A) *In vitro* neutralization. Hemocytes infected with WSSV or WSSV plus WBD were collected to identify virus infection. hpi: hours post-infection. (B) *In vivo* neutralization.

The neutralization experiments were performed *in vitro* and *in vivo* to identify whether the WBD (Fig. 2) on Glut1 can bind to WSSV and then plays important roles in WSSV infection. Because the protein with many transmembrane regions was difficult to produce and purify, the synthetic peptides, LSKYSYSL, containing WBD was applied in this study. The expression of WSSV immediate early gene ie1 was used as WSSV infection indicator because *ie1* could be detected at 2–4 h post-infection [19], [20]. EF1-α was set as experiment control [21]. Because the stable shrimp cell line for WSSV infection assay is lacking, the *in vitro* neutralization experiment was performed with primary cultured hemocytes firstly to check the virus binding ability of WBD. Due to the limitation of primary culture, the sampling times of the *in vitro* neutralization experiment was limited in 20 h. In Fig. 8A, the result of RT-PCR showed that WSSV infection can be detected in hemocytes from 6 h after infection. However, the expression of WSSV *ie1* can not be detected in the cells infected with WSSV treated with WSD peptides. The result clearly indicated that the WBD peptide can bind to WSSV, and then inhibit WSSV enter hemocytes.

The further neutralization experiment was performed *in vivo* in shrimps. As shown in Fig. 8B, the mortality increased steadily from 3.5 day to 10.5 day in the group challenged with WSSV (positive control). Although the mortality in the group challenged with WSSV plus WBD peptide (10 µg/g shrimp) was similar to positive control group, the mortality in the groups challenged with WSSV plus WBD peptide (20 µg/g shrimp) were lower than WSSV infection groups. IQ2000 WSSV Detection and Prevention System confirmed that shrimp mortality was due to WSSV infection (data not shown). The result in this experiment indicated that the WBD peptide can delay the mortality in shrimps upon WSSV challenge.

## Discussion

To infect a target cell, a virion proceeds through a multistep entry process, during which each step is preprogrammed and tightly regulated in time and space [6]. Considerable progress has been made in determining the mechanisms of cell entry used by enveloped RNA viruses, but less is known about this process for enveloped DNA viruses. RNA viruses typically have a single transmembrane (TM) glycoprotein that undergoes a conformational transition triggered by receptor recognition or low pH, leading to the insertion of a fusion peptide into the plasma membrane or the membrane of an endocytic vesicle [22], [23]. Differing from RNA viruses, the basic fusion machinery of the Herpesviridae, a family of large enveloped DNA viruses, includes three glycoproteins as well as subfamily-specific receptor binding proteins [24]. Eight TM proteins were found to be required for entry of vaccinia virus (VACV) [25]. It indicated that the enveloped DNA viruses may utilize a protein complex to deal with virus entry.

To date, it is not very clear how white spot syndrome virus (WSSV), also a kind of large enveloped DNA virus, enters the host cell. In our previous study, *Penaeus monodon* chitin-binding protein (PmCBP), a cell membrane protein, was found to at least interact with eleven envelope proteins (VP24, VP32, VP39B, VP41A, VP53A, VP53B, VP51B, VP60A, VP110, VP124 and VP337). The neutralization experiments only with PmCBP or VP53A significantly, but incompletely, blocked the WSSV infection [10]. It indicated that there was also a viral envelope protein complex dealing with WSSV entry, similar to the herpesvirus and vaccinia virus. Our result did not exclude the possibility that additional viral and/or host proteins participate in the virus entry complex. Thus, in this study, another host cell membrane protein, glucose transporter 1 (Glut1), was screened and identified to partake in the complex. The results of Y-2-H and far western blot firmly suggested that Glut1 could both interact with VP53A and PmCBP. We speculate that host cell membrane proteins Glut1 and PmCBP may form a heterodimer structure which could interact with the WSSV envelope complex formed by the eleven envelope proteins.

Transporters could be the receptors for some viruses. Recent studies have determined that glucose transporter 1 (GLUT-1) is required in the entry of human T-cell lymphotropic viruses (HTLVs). Human Cytomegalovirus activates glucose transporter 4 expression in mammalian cells to increase glucose uptake during infection [26]. For HTLV-1, GLUT-1 facilitates entry at a step after the initial attachment to the target cell [27]. The cell surface receptor FLVCR for feline leukemia virus C (FeLV-C) is identified as a member of the major facilitator superfamily (MFS) and demonstrated to export cytoplasmic heme [17], [28]. Similarly, cell-surface receptors for gibbon ape leukemia virus (Glvr-1) and murine amphotropic retrovirus (Ram-1) are inducible sodium-dependent phosphate symporters [11]. It indicates that some retroviruses utilize related transporters for cell entry, and the molecule the transporter transports is important for cell survival. Although WSSV is not classified to *Retroviridae* and the precise role of Glut1 is still unknown, by reviewing the studies of retroviruses, we can speculate that the biological role of Glut1 must be very important and the cell death after WSSV infection must be in connection with Glut1.

Tissue tropism analysis by RT-PCR showed that *Glut1* was constitutive and highly expressed in pleopod, gill, stomach, midgut, heart, lymphoid organ, nervous tissue, hepatopancreas, and hemolymph (Fig. 4). Review in the research about histopathology and tissue tropism after WSSV infection, the virus mainly infects cells in tissues of ectodermal (cuticular epidermis, fore- and hindgut, gills, and nervous tissue) and mesodermal (lymphoid organ, antennal gland, connective tissue, and hematopoietic tissue) origins, whereas tissues of endodermal origin (hepatopancreatic tubule epithelium and midgut epithelium) are refractory to WSSV infection [29], [30], [31]. The tissue tropism of *Glut1* coincided with the tissues/organs which WSSV infected. Besides, the research of FLVCR, the receptor for FeLV-C, has already revealed that virus binding to the hematopoietic cells induced aplastic anemia because the transporter function was affected [17]. In the late stage of WSSV infection, the amount of hemocytes in the infected shrimp was dramatically reduced and most tissues and organs showed massive multifocal necrosis [2]. Although the real function of Glut1 needs to be identified, we propose that the transporter function of Glut1 might be affected by WSSV and it leads to cell death.

In this study, the synthetic WSSV binding domain (WBD) peptide can completely inhibit WSSV infection *in vitro*, but promote only 25% survival rate of the shrimps which were challenged by WSSV in the *in vivo* neutralization experiment. This may be because the short synthetic peptide, without any protection, might easily degrade in the animal body, or the partial peptide can not present the protective effect due to its protein conformation. Nevertheless, this result indeed provides a direction to develop efficient antiviral strategies or therapeutic methods using WBD.

In conclusion, a glucose transporter was revealed to join in the virus entry complex. Up to the present, it is still unclear how and when the protein complex forms. Our evidence does not exclude the possibility that additional or alternative WSSV receptors and receptor binding proteins occur. To date, besides PmCBP and Glut1, more than one shrimp protein were supposed to mediate WSSV infection [7], [9]. The antiviral strategies on the basis of interrupting the binding between shrimp and viral structural proteins were applied to protect shrimps from virus infection [32], [33]. However, the results of this study provide directions for further research.

## Materials and Methods

### Yeast two-hybrid (Y-2-H) assay

The plasmids, yeast strains and library construction kits used in this experiment were all adopted from BD Matchmaker library construction and screening kits (BD Biosciences Clontech). The construction of bait plasmid pGBK-VP53A which contained the GAL4 DNA-binding domain (BD) and the prey library which contained activation domain (AD), and the screening procedure, were as described in previous yeast two-hybrid assay [8]. In this study, pGBK-VP53A and pGAD-PmCBP were regarded as positive controls because their interaction has been confirmed. Negative controls employed pGBKT7 only (BD) and pGADT7-Rec only (AD). In this study, a positive clone Y455 was identified and named as glucose transporter 1 (Glut1) after sequence alignment. The interaction between VP53A and the gene product form clone Y455 was confirmed by far western blotting and the materials and methods for far western blotting in this study were summarized in the later section.

### Identification of the full-length Glut1 transcript

The sequences of the primers used in this study are shown in Table 1. Total RNA isolation from the pleopod of *Litopenaeus vannamei* was as described previously [8] with the following modification: Briefly, for the isolation of total RNA, tissues (100 mg) were homogenized in 1 ml TRIzol reagent (Invitrogen) and then subjected to 2-propanol extraction and ethanol precipitation according to the manufacturer's recommendations. Total RNA in 75% ethanol was centrifuged at 14,000 g for 30 min at room temperature. The pellet was resuspended in DEPC-water and quantified by spectrophotometer.

**Table 1:** List of primers used in this study.

Primer	Sequence (5′-3′)	Usage
Glut1-5′RACE-sp1	GTAGCACAGACTCTGGCCGCTGA	*Glut1* 5′RACE
Glut1-5′RACE-sp2	CAAGGAGTAACTGTACTTAGAC	*Glut1* 5′RACE
LvGlu1-F	CAAGGGACCATCCGCTTGAACA	*L. vannamei Glut1* Gene cloning
LvGlu1-R	GTCAGCCACACATGACGAAGCGG	*L. vannamei Glut1* Gene cloning
Glut1-RT-F	AGGGGATAGGCACCATGCAGAC	*Glut1* RT-PCR
Gluct1-RT-R	AAGGCGCGTCGCCTCGAAGG	*Glut1* RT-PCR
actin-RT-F	CCGTCATCAGGGTGTGATGGT	*β-actin* RT-PCR
actin-RT-R	CCACGCTCAGTCATGATCTTCA	*β-actin* RT-PCR
Glut1-*Bam*HI-F1	ACTTGGATCCGATGTTCATAACCCTGACGCCCCC	Recombinant protein producing
Glut1-*Hind*-R1	GCGAAAGCTTCTAGGCAGGCCGCCAGTCCTCGT	Recombinant protein producing
wssv ie1-F	GACTCTACAAATCTCTTTGCCA	WSSV RT-PCR
wssv ie1-R	CTACCTTTGCACCAATTGCTAG	WSSV RT-PCR
EF1-α-F	TGCCCTGGACAACATCGAGC	EF1-αRT-PCR
EF1-α-R	CGGGCACTGTTCCAATACCT	EF1-αRT-PCR

For the mapping of 5′ terminus of the *Glut1* transcript, the reaction was determined by rapid amplification of the cDNA 5′ end (5′ RACE) using a commercial FirstChoice® RLM-RACE kit (Ambion) according to the instructions provided by the manufacturer. Briefly, an aliquot of 10 µg RNA was treated with Calf Intestine Alkaline Phosphatase (CIP) at 37°C for 1 h to remove free 5′-phosphates from RNA and then re-extracted with phenol-chloroform. The CIP-treated total RNA was then treated with Tobacco Acid Pyrophosphatase (TAP) at 37°C for 1 h to remove the cap structure from full-length mRNA, leaving a 5′ monophosphate. Then, a 45 base RNA adapter oligonucleotide supplied by the kit was ligated to the 10 µg TAP-treated RNA. The first strand cDNA was synthesized by the addition of 2 µl 10×RT buffer, 2 µl random decamers, 4 µl dNTP mix, 1 µl RNase inhibitor, and 1 µl M-MLV reverse transcriptase. The reaction proceeded at 50°C for 1 h. The PCR for *Glut1* was performed using the primer Glut1-5′RACE-sp1 and 5′RACE Outer Primer supplied by the kit for the 1-step PCR reaction and Glut1-5′RACE-sp2 and 5′RACE Inner Primer supplied by the kit for the 2-step PCR reaction. The design of Glut1-5′RACE-sp1/Glut1-5′RACE-sp2 primer set was based upon the obtained partial cDNA sequence. Finally, LvGlu1-F/LvGlu1-R primer set (Table 1) based upon the obtained full-length of Glut1 sequence to double-check the accuracy of the Glut1 full-length sequence in *L. vannamei*. The final products were characterized by subcloning and sequencing, and the resulting sequences were then analyzed and compared with other sequences. The sequence data was deposited to GenBank (GenBank accession no.: HQ620544) and will be released after the manuscript is accepted.

### Tissue tropism analysis of Glut1 by RT-PCR

The organs – pleopod, gill, stomach, midgut, heart, lymphoid organ, nervous tissue, hepatopancreas, and haemolymph - were collected form *L. vannamei*. Total RNA was isolated as described in Materials and methods. After RNA extraction, an aliquot of 10 µg RNA was treated with 200 U of RNase-free DNase I (Roche) at 37°C for 30 min to remove any viral genomic DNA contamination and then re-extracted with phenol-chloroform. The DNase-treated total RNA was denatured by heating at 65°C for 5 min in 10 µl DEPC-water containing 50 µM oligo dT-anchor primer (Roche) and 1 µl 10 µM dNTP mix. The first strand cDNA was synthesized by the addition of 2 µl 10×RT buffer, 2 µl 0.1 M DTT, 4 µl 5 mM MgCl_2_, 1 µl 40 U RNaseOUT (Invitrogen), and 1 µl 200 U Superscript III reverse transcriptase (Invitrogen). The reaction proceeded at 50°C for 50 min, and was terminated at 85°C for 5 min. Then, the cDNA was treated with 1 µl RNase H to remove the RNA template from the cDNA.

Further, for the PCR reaction, the appropriate gene specific primers, Glut1-RT-F/Glut1-RT-R for *Glut1* and actin-RT-F/antin-RT-R for *β-actin* (Table 1), were then used for PCR reaction. The products were resolved in 1% agarose gel.

### Recombinant Glut1 over-expression in *Esherichia coli* and antiserum production

The DNA fragment encoding the extracellular portion between TM1 and TM2 of Glut1 was amplified from shrimp *L. vannamei* cDNA by PCR with the Glut1-*Bam*HI -F1/Glut1-*Hind*-R1 primer set (Table 1) for 40 cycles of denaturation at 94°C for 30 s, annealing at 55°C for 30 s, extension at 72°C for 50 s, and then a final extension at 72°C for 5 min. The PCR product was ligated to pGEM®-T Easy (Promega) plasmid, and then the resultant plasmid was sequenced by a commercial service (Mission Biotech Co., Ltd, Taiwan). After confirming the sequence, the resultant plasmid, pGEM-T-Glut1 was cleaved with *Bam*HI and *HindI*III, and the amplified fragment was then cloned to pET28b+ (Novagen) at *Bam*HI and *Hind*III sites. The resultant recombinant plasmid, pET28-Glut1 was transformed into *E. coli* BL21 (DE3) strain. *E. coli* BL21 (DE3) cells were cultured in LB medium with 25 mg/ml kanamycin and the protein was induced with 1 mM isopropyl-β-D-thiogalactopyranoside (IPTG). The recombinant Glut1 proteins tagged with 6 consecutive histidines (6×His-tagged Glut1) were purified using QIAexpressionist™ nickel-nitrilotriacetic acid (Ni-NTA) metal-affinity chromatography (Qiagen) according to the manufacturer's recommendations. Finally, the eluted protein was then concentrated using Amicon Ultra-15 centrifugal filters (Millipore) in PBS buffer and stored at 4°C for further antiserum production and far western assay.

New Zealand white rabbits were hyperimmunized by injection with 250 µg proteins emulsified in complete Freund's adjuvant. Subsequent booster injections were carried out with 250 µg protein emulsified in incomplete Freund's adjuvant. The antiserum was collected after the antibody titer had peaked.

### Yeast and shrimp tissue lysate

The yeast and heart tissue of shrimp were homogenized with SDS lysis buffer (1% SDS, 0.01 M EDTA, 0.05 M Tris-HCl, pH 8.0). The homogenate was centrifuged at 10,000×g for 5 min and then supernatant containing the total protein was transferred to new tube. The protein content was estimated using Bradford assay.

### Western blot analysis

For Western blotting analysis, yeast lysates, shrimp tissue lysate or recombinant protein that had been separated in SDS-PAGE was transferred onto a polyvinylidene difluoride (PVDF) membrane (Millipore). Membranes were blocked in 5% skim milk (Difco Laboratories) and 1% Tween20 in TBS buffer (50 mM Tris-HCl and 0.5 M NaCl, pH 7.5). Immunodetection was performed by incubation of the blot in a polyclonal rabbit anti-HA antibody, anti-Glut1 antibody, anti-PmCBP antibody or pre-immune antiserum diluted 1∶2000 in TBST with 5% skim milk for 1 h at room temperature. Subsequently, goat anti-rabbit IgG antibody conjugated with horseradish peroxidase (Jackson) was used at a concentration of 1∶5000 and detection was performed with a Western Lightning™ Plus-ECL (Bioman).

### Cellular localization of Glut1 by indirect immunofluorescence assay

Hemolymph was collected from the shrimp *L. vannamei* using a syringe that contained cold anti-coagulant (27 mM sodium citrate, 336 mM NaCl, 115 mM glucose and 9 mM EDTA, pH 7.0). The hemocytes were collected by centrifugation at 200 g at 4°C for 10 min, and then resuspended in 2×Leibovitz's L-15 (Invitrogen) containing 20% fetal bovine serum (FBS), 2 g/l glucose, 100 UI/ml penicillin, 100 µg/ml streptomycin and 2.4% NaCl. The hemocytes were incubated at room temperature on microscope slide for 2–3 hour, and monolayer cells formed. The monolayer hemocytes were rinsed three times with 2×L-15 medium, and then were fixed in paraformaldehyde (4% in PBS) for 10 min at 4°C. After fixation, the hemocytes were washed three times with PBS, and incubated with blocking buffer (5% bovine serum albumin and 5% normal goat serum in PBS) for 1 h at room temperature. The hemocytes were then treated with polyclonal rabbit anti-Glut1 antibody and preimmune serum (1∶200 in blocking buffer) for 1 h at room temperature. Next, the cells were washed three times with PBST (0.2% Tween-20 in PBS) and reacted with Cy3-conjugated goat anti-rabbit IgG antibodies (1∶500 in PBS; Jackson ImmunoResearch) at room temperature. Counterstaining of the nucleus was performed with DAPI. After being washed three times with PBST, the cover glasses were wet mounted and the fluorescent signals were examined with a Leica TCS SP5 Confocal Spectral Microscope Imaging System.

### Far western blot analysis

In this study, different far western blot analyses were performed. (1) In confirming the interaction between VP53A and the gene product form clone Y455, the yeasts transformed with pGAD-Y455 or pGADT7-Rec were separated by SDS-PAGE, transferred onto a PVDF membrane and renatured gradually at 4°C overnight in HEPES buffer (20 mM HEPES, 100 mM NaCl, 1 mM EDTA, 1 mM dithiothreitol, 0.1% Tween20, 10% glycerol, pH 7.5) containing 5% skim milk. To determine whether VP53A interacts with the gene product form clone Y455, the blot was incubated with another 0.5 µg probe recombinant VP53A protein in incubation buffer (20 mM Tris/HCl, 150 mM NaCl, 0.05% Tween-20, 3% skim milk, pH7.5). Then the blot was incubated in a polyclonal rabbit anti-VP53A antibody diluted 1∶5000 in TBST with 5% skim milk for 1 h at room temperature. Subsequently, goat anti-rabbit IgG antibody conjugated with horseradish peroxidase (Jackson) was used at a concentration of 1∶5000 and detection was performed with a Western Lightning™ Plus-ECL (Bioman). The production of recombinant VP53A and the generation of polyclonal rabbit anti-VP53A antibody were as mentioned in the previous studies [8], [10]. In this experiment, the yeast transformed with pGADT7-Rec only was set as control. (2) In confirming the interaction between Glut1 and PmCBP, recombinant proteins Glut1 or PmCBP containing His tag were separated by SDS-PAGE, transferred onto a PVDF membrane and also renatured gradually at 4°C overnight in HEPES buffer containing 5% skim milk. To determine whether Glut1 interacts with PmCBP, the blot was incubated with another 0.5 µg probe recombinant PmCBP or Glut1 proteins in incubation buffer. Then the blot was incubated in a polyclonal rabbit anti-PmCBP or anti-Glut1 antibodies diluted 1∶5000 in TBST with 5% skim milk for 1 h at room temperature. The further steps were as described above. The production of recombinant PmCBP and the generation of polyclonal rabbit anti-PmCBP antibody were as mentioned in the previous study [10]. In this experiment, the short His tag containing protein purified from *E. coli* transformed with pET28 only was set as control.

### *In vitro* neutralization

The peptide (LSKYSYSL) was synthesized by a manufacturer (Seeing Bioscience Co., Ltd, Taiwan) with a purity of 75% and the sequence was confirmed by mass spectrometry. The method of hemocyte collection was as described earlier. WSSV (10^6^ copies) was incubated with synthetic WBD peptides or PBS, as a control group, for 30 min at room temperture. The mixtures of peptides and WSSV were then incubated with the hemocyte (4.0×10^5^ cells) at 28°C in a 24-well microplate. After incubation for 2 h, 6 h and 20 h, haemocytes were washed with PBS and proceeded to RT-PCR. The expression of WSSV immediate early gene *ie1* was used as WSSV infection indicator and *EF1-α* was set as control (Table 1). The experiments were conducted at least 3 times with both peptide-treated and control groups.

### *In vivo* neutralization

This study was carried out by using invertebrate species *L. vannamei*. According to the Guide for Animal Use Protocol of the Institutional Animal Care and Use Committee (IACUC) of National Taiwan Ocean University, ethics approval is not required. The shrimp *L. vannamei* (body length, 5±1 cm; weight, 3±0.5 g) used in the experiment was obtained from the Aquatic Animal Center, National Taiwan Ocean University. Shrimps were acclimatized in the laboratory for about 1 week before the experimentation. The experimental shrimps were then further divided into four groups, with three replicates of 10∼15 shrimps in each group. The shrimps were injected as follows: group 1 with WSSV (10^6^ copies) as the positive control, group 2 with PBS as the buffer negative control, group 3 with WSSV (10^6^ copies) plus WBD peptide (10 µg/g shrimp) and group 4 with WSSV (10^6^ copies) plus WBD peptide (20 µg/g shrimp). The synthetic peptide was mixed with virus at room temperature for 1 h and then injected into the experimental shrimps. During the acclimatization and experimental period, shrimp were fed twice daily with a commercial shrimp diet. The water conditions were kept at 25±1°C and a salinity of 30‰. During the experimental period, the shrimp mortalities in every group were recorded 2 times a day. Cumulative mortality was calculated and subjected to paired sample t-test. Differences were considered significant at p<0.05.
